# Stigmatizing Attitudes Toward Depression Among Male and Female, Medical and Non-medical Major College Students

**DOI:** 10.3389/fpsyg.2021.648059

**Published:** 2021-06-25

**Authors:** Haoyu He, Qiuxia Wu, Yuzhu Hao, Shubao Chen, Tieqiao Liu, Yanhui Liao

**Affiliations:** ^1^Department of Psychiatry, The Second Xiangya Hospital, Central South University, Changsha, China; ^2^National Clinical Research Center for Mental Disorders, Changsha, China; ^3^Department of Psychiatry, Sir Run Run Shaw Hospital, School of Medicine, Zhejiang University, Hangzhou, China

**Keywords:** depression, stigma, social distance, college students, gender differences, medical and non-medical students

## Abstract

**Background**: Stigma is often reported to be a barrier to the treatment and rehabilitation of depression. However, little is known about stigma toward people with depression among college students in China.

**Methods**: Using a questionnaire with a case vignette describing depression, a total of 1,056 students from nine colleges/universities in Hunan Province of China were included in this study. The questionnaire addressed the attitudes toward depression and the desire for keeping a distance from depressed individuals. The current study explored the stigma attitudes of college students toward people with depression and the desire for social distancing, as well as the gender (male and female) and major (medical and non-medical) differences.

**Results**: Over half of the respondents agreed that people described in the vignette were “dangerous” (60.7%) and “could snap out of the problem” (58.7%). Compared with female students, males were more likely to agree that “If I had this problem, I would not tell anyone” (7.0 vs. 13.2%, *p* = 0.001); compared with non-medical students, medical students were more likely to agree that “The problem is a sign of personal weakness” (38.0 vs. 50.0%, *p* < 0.001). A lot of respondents would be unwilling to “marry into the family of people with depression” (71.1%) or “work closely with them” (45.1%). Compared with male students, females were more unwilling to “work closely with them” (40.3 vs. 47.5%, *p* = 0.026).

**Conclusion**: This study found that a high proportion of Chinese college students showed stigma toward and desire for social distancing from people with depression, male students and medical major students showed higher stigma in some subscale items toward people with depression. The present results suggest that more anti-stigma interventions should be applied for Chinese college students to help prevent or reduce stigma attitudes toward people with depression.

## Introduction

Depression (major depressive disorder or clinical depression), a leading cause of disability worldwide, is one of the most prevalent mental health disorders that affects more than 300 million people worldwide (Marcus et al., 2012). The prevalence of 12-month depression (weighted) in the general Chinese population was 3.6% (Huang et al., 2019), and the overall prevalence of depression among Chinese university and college students was 23.8% (Lei et al., 2016). Depression in university students negatively affects psychological and physical health, such as sleep disturbances (Eller et al., 2006), academic struggles (Abu Ruz et al., 2018), self-injury and suicidal thoughts (Bayram and Bilgel, 2008), smartphone addiction (Matar Boumosleh and Jaalouk, 2017), and heavy episodic drinking (Mushquash et al., 2013). Furthermore, youth depression is a major risk factor for suicide (Majeed and Lee, 2017). University students experience depression at a significantly higher rate than the general population, and also higher than their non-college contemporaries (Bayram and Bilgel, 2008; Lei et al., 2016; Gao et al., 2020). However, negative attitudes and beliefs (stigma) toward people with depression are common. For example, a community-based sample study in China showed that 53.0% participants reported personal stigma, and 83.4% reported perceived stigma (Yang et al., 2020); a survey in Japan reported that 30.7% of the participants thought that a weak personality causes depression (Yokoya et al., 2018).

Stigma refers to the process of distinguishing and labeling individuals with certain socially undesirable characteristics that are different from the norms of society (Goffman, 2009). Stigma toward mental illness (such as depression) is usually associated with violence, crime, abnormality, or general dangerousness, which results in factual separation of stigmatized individuals through loss of status, economic, and political power (Anderson et al., 2015). University students are in a critical developmental period during which they are separated from family, develop new social connections, increase their autonomy and responsibility, and create more self-awareness and control (Duffy et al., 2019). Moreover, well-educated university students are important resources for the development of science, technology, and economic construction in the country. Their attitude and view toward depression may generate profound and long-term effects on themselves and to the society as a whole. A research found that depressed university students are more sensitive toward stigma from classmates, schoolmates, teachers, and caregivers (Cheng et al., 2013). Under the negative influence of stigma, they might worry that their depression is prone to be revealed by mental health service utilization (Guille et al., 2010). Moreover, there were higher levels of stigma among university students regarding mental health treatment, and only a minority of depressed young students sought professional help for treatment (Eisenberg et al., 2009; Ibrahim et al., 2019). However, the more they fear stigma (perceived public stigma and personal stigma), the more resistant they are to seek professional assistance voluntarily, and the worse their depression becomes, which forms a vicious circle (Lauber and Rössler, 2007). Stigma is also considered an obstacle for the public to obtain an objective understanding, and as a rational attitude toward depressed university students, accelerating the formation of this vicious circle.

College- and university-level stigma is not only negatively associated with self-reported of suicidal thoughts and self-injury, but also negatively associated with medication utilization, clinic visits, and informal support (Gaddis et al., 2018). Stigma attitudes toward depression even prevalent among medical students (Cheng et al., 2013). However, young people with medical background were more unwilling to seek mental health treatment (Guille et al., 2010). A survey of 194 medical students from the School of Medicine at the University of California, San Francisco, reported that only 22% depressed students were willing to receive mental health counseling services (Givens and Tjia, 2002). Moreover, the stigmatization from medical students affected patients with depression in the quality of care offered to patients and caused self-stigmatization and other related consequences (Suwalska et al., 2016). Exploring the misunderstanding and stigma toward depressed youth among medical and non-medical students can provide insights to overcome these barriers and improve this underserved population’s mental health.

The aim of this study was to explore college/university students’ stigma attitudes toward people with depression and the desire for social distancing, as well as the gender (male and female) and major (medical and non-medical) differences. Considering the high level of stigma attitudes toward depression in Chinese community (Yang et al., 2020), and the majority of medical youth unwilling to seek professional help (Guille et al., 2010; Gaddis et al., 2018), this study hypothesized that (i) stigma attitudes toward depression is prevalent among college/university students; (ii) compared with the non-medical counterparts, medical students show higher level of stigma attitudes toward depression; (iii) male students show greater preferred social distancing than female students toward people with social anxiety disorder (Anderson et al., 2015); and (iv) men showed more stigmatization than women for depression and other mental illness (Korszun et al., 2012). However, the gender difference in stigma toward depression is largely unknown among university students. Thus, it is also hypothesized that male students keep more stigma attitudes toward depression than females. We assessed the gender and medical and non-medical major differences in college students’ stigma attitudes toward depression and the desire for social distancing.

## Materials and Methods

### Procedures

The protocol was approved by the ethics committee of the Second Xiangya Hospital of Central South University (No. S095, 2013). The convenience sampling method was used in the present study. Considering the representativeness of the sample, this study randomly selected different classes by school, grade, and major. Nine colleges/universities in Hunan (a province in southern China) with different school levels and majors were selected. The aim of the present study was explained in the questionnaires, and oral informed consent was obtained from all the respondents. They were encouraged to independently analyze the vignette and answer a battery of questions, including demographic information, depression stigma scale (DSS), and social distance scale (SDS).

### Sample

Data were collected from nine colleges/universities in Hunan Province of China from September 2014 to January 2015, including three national key universities (Project 985 and 211): Central South University, Hunan University, and Hunan Normal University; four ordinary universities: Hunan University of Chinese Medicine, Central South University of Forestry and Technology, Hunan University of Technology and Business, and Changsha Medical University; and two 3-year colleges: Changsha Society Work College and Hunan College of Information. NCSS_PASS 2011 software^1^ was used to calculate the sample size. According to our previous studies (Wu et al., 2017; Hao et al., 2020), the required sample size in the current study was 1,220 participants. The sample size was calculated as the proportion of the stigma to 50%, at the 95% confidence level with a 6% CI width (3% marginal error) and a 10% non-response rate. A total of 1,224 participants (136 students in each school) were invited to complete the face-to-face paper and pencil-based questionnaires independently.

### Measurements in the Survey Questionnaires

The questionnaires consisted of four parts: the first part was a brief introduction to the study, including the research sponsor, purpose, method, and significance; the second part was a range of questions about demographic data; the third part was a vignette describing a person with symptoms of depression – these symptoms were appropriately locally adapted from the questionnaire developed by Jorm et al. (1997), and met with the diagnostic criteria in Diagnostic and Statistical Manual of Mental Disorders, Fourth Edition (DSM-IV), and the International Classification of Diseases, Tenth Revision (ICD-10); and the last part included scales of depression stigma and social distance based on the context of the vignette, including DSS and SDS.

#### Depression Stigma Scale

The DSS contains personal stigma subscales (nine items) and perceived stigma subscales (nine items). The statements in each item of the two subscales are the same with the exception of the subject of items. In the personal stigma subscales, respondents were asked their own attitude toward people with depression symptoms described in the vignette (e.g., “People with depression could snap out of it if they wanted”). In the perceived stigma subscales, respondents were asked their beliefs about most of the other people’s attitudes toward people with depression symptoms described in the vignette (e.g., “Most people believe that people with depression could snap out of it if they wanted”). The response of each item was measured on a five-point scale ranging from “strongly agree” to “strongly disagree” (Griffiths et al., 2004). The Chinese version of the scale has been widely used with good reliability and validity (Zhu et al., 2019; Yang et al., 2020).

#### Social Distance Scale

The five-item short measurement of SDS was developed by Link et al. (1999) to measure the desire for social distancing from the person with mental illness. The Chinese version of the SDS was used to estimate the willingness to come into contact (such as live next door, marry into family) with the person described in the vignette. The response of each item was measured on a four-point scale, which ranged from “definitely willing” to “definitely unwilling.” The reliability and validity of its Chinese version has been tested, and the results showed that all the indicators met the requirements of psychometrics (Haoyu et al., 2020).

### Statistical Analysis

All data were analyzed by SPSS 22 (IBM SPSS, Armonk, NY, United States) and Excel. Descriptive statistics were applied for demographic data (percentage), stigma attitudes toward people with depression (percentage frequencies and 95% CI) and social distance (percentage frequencies and 95% CI). The options of “agree” and “strongly agree” were combined into one option on the DSS, and the options of “definitely unwilling” and “probably unwilling” were combined into one option on the SDS. The combined options represent the positive and negative attitudes of the respondents. Pearson’s Chi-square test was used to assess the significant difference in each item on the DSS and SDS among different demographic variables (gender, major, educational level, and school level) in the proportion of agreement. Multiple linear regression analysis was applied to explore any stigma or social distance–associated factors, including education level (1 = graduate students, 2 = postgraduate students); major (1 = non-medicine, 2 = medicine); school level (1 = national key university, 2 = ordinary universities, and 3-year college); age and gender (1 = male, 2 = female). The value of *p* was set at <0.05 for statistical significance.

## Results

A total of 1,056 (the response rate: 86.3%) qualified questionnaires were included in the final analysis from 1,224 distributed questionnaires. Demographic characteristics are shown in Table 1. The average age of the respondents was 21.05 ± 2.54 (mean ± SD). The ratio of gender (male 33.62%: female 66.38%) was approximately 1:2, which may be resulted from the imbalance in gender ratio in many Chinese colleges and universities (Schwenk et al., 2010). In order to explore the stigma attitudes toward depression between medical and non-medical students, the respondents from nine majors were classified into non-medicine (71.2%) and medicine (28.8%) majors.

**Table 1:** Demographic characteristics of participants.

Participant characteristics	*N* = 1,056	
	*n*	%
Gender		
Male	355	33.62
Female	701	66.38
Marital status		
Married	27	2.56
Unmarried	1,029	97.44
Major		
Non-medicine	752	71.21
Medicine	304	28.79
Educational level		
Graduate students	888	83.91
Postgraduate students	168	16.91
University level		
National key university	463	43.84
Ordinary universities	402	38.07
Three-year college	191	18.09

### Stigma

The gender differences and medical and non-medical major differences in the percentage of participants who held stigma attitudes toward the person with depression in the vignette are shown in Table 2. Over half of the respondents agreed that people described in the vignette were “dangerous” (60.7%) and “could snap out of the problem” (58.7%). Compared with female students, males were more likely to agree that “If I had this problem, I would not tell anyone” (7.0 vs. 13.2%, *p* = 0.001); compared with non-medical students, medical students were more likely to agree that “Problem is a sign of personal weakness” (38.0 vs. 50.0%, *p* < 0.001).

**Table 2:** Percentage of participants who “agree” or “strongly agree” with statements about their attitudes toward the person in the vignette.

Statement about personal belief (DSS)	Total (*N* = 1,056)		Gender					Major				
	*n*	%	Male (*n* = 355)		Female (*n* = 701)		*p* ^*^	Non-medical (*n* = 752)		Medical (*n* = 304)		*p* ^*^
			*n*	% (95% CI)	*n*	% (95% CI)		*n*	% (95% CI)	*n*	% (95% CI)	
1. The person could snap out of the problem	620	58.7	216	60.8 (55.7–65.3)	404	57.6 (53.9–61.3)	0.316	459	61.0 (57.5–64.5)	161	53.0 (47.4–58.6)	0.016
2. Problem is a sign of personal weakness	438	41.5	150	42.3 (37.2–47.4)	288	41.1 (37.5–44.7)	0.716	286	38.0 (34.5–41.5)	152	50.0 (44.4–55.6)	**<0.0001**
3. Problem is not a real medical illness	316	29.9	111	31.3 (26.5–36.1)	205	29.2 (25.8–32.6)	0.498	238	31.6 (28.3–34.9)	78	25.7 (20.8–30.6)	0.054
4. People with this problem are dangerous	641	60.7	205	57.7 (52.6–62.8)	436	62.2 (58.6–65.8)	0.162	453	60.2 (56.7–63.7)	188	61.8 (56.3–67.3)	0.629
5. Avoid people with this problem	48	4.5	16	4.5 (39.8–50.2)	32	4.6 (42.4–49.7)	0.966	40	5.3 (3.70–6.90)	8	2.6 (0.8–4.4)	0.058
6. People with this problem are unpredictable	308	29.2	104	29.3 (24.6–34.0)	204	29.1 (25.7–32.5)	0.948	218	29.0 (25.8–32.2)	90	29.6 (24.5–34.7)	0.842
7. If I had this problem, I would not tell anyone	96	9.1	47	13.2 (9.7–16.7)	49	7.0 (5.1–8.8)	**0.001**	77	10.2 (8.0–12.4)	19	6.3 (3.6–9.0)	0.041
8. I would not employ someone with this problem	293	27.7	102	28.7 (24.0–33.4)	191	27.2 (23.9–30.5)	0.611	203	27.0 (23.8–30.2)	90	29.6 (24.5–34.7)	0.391
9. I would not vote for a politician with this problem	448	42.4	148	41.6 (36.5–46.7)	300	42.8 (39.1–46.5)	0.731	311	41.4 (37.9–44.9)	137	45.1 (39.5–50.7)	0.269
DSS total score (mean ± SD)	26.91 ± 3.56		27.05 ± 3.72		26.84 ± 3.52		0.378	27.06 ± 3.683		26.54 ± 3.321		**0.035**

CI, confidence interval; DSS, depression stigma scale. Data are n, % or mean ± SD.

* *p* < 0.05. *Bold value means p* < 0.05.

Supplementary Table 1 shows the under- and post-graduate differences, and school-level (national key/ordinary/3-year college) differences in the percentage of participants who held sigma attitudes toward the person with depression in the vignette. Supplementary Table 2 shows the under- and post-graduate differences, and school-level (national key/ordinary/3-year college) differences. Supplementary Table 3 shows the entire sample, gender differences, and medical and non-medical major differences in the percentage of participants who thought that other people may hold stigma attitudes toward the person with depression in the vignette.

### Social Distance

The gender differences and medical and non-medical major differences in the percentage of participants who were unwilling to have contact with the person described in the vignette are shown in Table 3. Many respondents were unwilling to “marry into the family of people with depression” (71.1%) or “work closely with them” (45.1%). Compared with male students, females were more unwilling to “work closely with them” (40.3 vs. 47.5%, *p* = 0.026). There were no other gender or medical and non-medical major differences in social distancing for people with depression.

**Table 3:** Percentage of participants who “probably unwilling” or “definitely unwilling” to have contact with the person described in the vignette.

Statement about personal belief (SDS)	Total (*N* = 1,056)		Gender					Major				
	*n*	%	Male (*n* = 355)		Female (*n* = 701)		*p* ^*^	Non-medical (*n* = 752)		Medical (*n* = 304)		*p* ^*^
			*n*	% (95% CI)	*n*	% (95% CI)		*n*	% (95% CI)	*n*	% (95% CI)	
1. Live next door	289	27.4	103	29.0 (24.3–33.7)	186	26.5 (23.2–29.8)	0.393	211	28.1 (42.9–31.3)	78	25.7 (20.8–30.6)	0.428
2. Spend the evening socializing	208	19.7	78	22.0 (17.7–26.3)	130	18.5 (15.6–21.4)	0.186	150	19.9 (17.0–22.8)	58	19.1 (14.7–23.5)	0.748
3. Make friends	253	24.0	93	26.2 (21.6–30.8)	160	22.8 (19.7–25.9)	0.225	172	22.9 (19.9–25.9)	81	26.6 (21.6–31.6)	0.193
4. Work closely	476	45.1	143	40.3 (35.2–45.4)	333	47.5 (43.8–51.2)	**0.026**	335	44.5 (40.9–48.1)	141	46.4 (40.8–52.0)	0.588
5. Marry into family	751	71.1	243	68.5 (63.7–73.3)	508	72.5 (69.2–75.8)	0.174	533	70.9 (67.7–74.1)	218	71.7 (66.7–76.8)	0.787
DSS total score (mean ± SD)	11.5 ± 2.76		11.59 ± 2.883		11.45 ± 2.701		0.439	11.46 ± 2.799		11.58 ± 2.674		0.536

CI, confidence interval; SDS, social distance scale. Data are n, % or mean ± SD.

* *p* < 0.05. *Bold value means p* < 0.05.

The under- and post-graduate differences, and school-level (national key/ordinary/3-year college) differences in the percentage of participants who were unwilling to have contact with the person with depression described in the vignette are shown in Supplementary Table 4.

### Predictors for Stigma and Social Distance

Predictors (education level, major, school level, age, and gender) for stigma and social distance are shown in Table 4. Compared with ordinary universities and 3-year colleges, students from national key universities are more likely to hold stigma attitudes toward depression and keep social distance toward people with depression. Also, compared with postgraduate students, graduate students were more likely to hold stigma attitudes toward depression. The analysis did not find any associations among major, age, and gender with the total score of stigma or social distance.

**Table 4:** Predictors for stigma and social distance by multiple linear regression analysis.

Dependent variable	Predictors	*B*	*t*	*p* ^*^	*R*	*R*^2^	*Adj.R*^2^
DSS total score	Education level	−2.713	−2.604	**0.009**	0.153	0.023	0.019
	Major	−0.213	−0.427	0.669			
	School level	−1.307	−3.804	**0.000**			
	Gender	0.012	0.024	0.980			
	Age	−0.060	−0.385	0.700			
SDS total score	Education level	−0.472	−1.201	0.230	0.101	0.010	0.006
	Major	−0.111	−0.592	0.554			
	School level	−0.303	−2.335	**0.020**			
	Gender	−0.153	−0.816	0.415			
	Age	−0.048	−0.818	0.413			

DSS, depression stigma scale; SDS, social distance scale.

* *p* < 0.05. *Bold value means p* < 0.05.

## Discussion

To the best of our knowledge, this is the first study that investigated the gender (male and female) and major (medical and non-medical) differences in Chinese college students’ stigma attitudes toward people with depression and the desire for social distancing by a case vignette. This study found that there is a high level of stigma and desire for social distancing among college students toward depressive people. This study also showed that male students and medical students hold higher stigma in some subscale items toward people with depression. To be specific, over 60% of respondents agreed that people described in the vignette were “dangerous” (60.7%) and “could snap out of the problem” (58.7%). Compared with female students (7.0%), male students (13.2%) were more likely to agree that “If I had this problem, I would not tell anyone”; compared with non-medical students (38.0%), medical students (50.0%) were more likely to agree that “the problem is a sign of personal weakness.” Over 70% respondents would be unwilling to “marry into the family of people with depression,” and more than 45% unwilling to “work closely with them.” Compared with male students (40.3%), females (47.5%) were more unwilling to “work closely with them.”

### Stigma

In this study, the most common stigmatizing attitudes toward people with depression among college students were “people with this problem are dangerous” and “the person could snap out of the problem.” Danger often has been labeled among individuals with depression. It is highly consistent with other studies (Link et al., 1999; Schwenk et al., 2010; Kashihara, 2015). A sample of university students in Qatar reported that over 60% of students believed that people with mental illness are dangerous (Zolezzi et al., 2017). A systematic review and meta-analysis reported that one-third of young people in India display “dangerous” and other negative attitudes toward people with mental health problems (Gaiha et al., 2020). A study compared perceptions of mental health among college students between two countries, and found that more college students from Vietnam tended to realize that individuals with mental illness were dangerous than their US counterparts (Kamimura et al., 2018). In this study, 61.8% of medical students and 60.2% of non-medical students held this view. Our previous study of 1,123 non-mental health professionals in six general hospitals in Hunan Province showed that more than 70% of them believed that people with depression are dangerous (Wu et al., 2017, 2019). Furthermore, a review indicated that this stigma is even widespread among mental health professionals (Schulze, 2007). In addition to beliefs of dangerousness, the current study found that perceived stigma was generally higher than personal stigma. Similar trends have been reported by other studies on university students from different countries, such as Australia and the United States (Busby Grant et al., 2016; Pompeo-Fargnoli, 2020). Dualistic thinking about mental disorders among university students may contribute to this view. Multilevel efforts are encouraged to overcome mental illness stigma, such as changing attitudes and practices among mental health professionals, formulating policies and regulations, alternating media depictions, and enhancing empathy (Hinshaw and Stier, 2008).

### Social Distance

The current study found that keeping social distance from depression patients is common among university/college students, which is consistent with the fact that the social distancing from people with mental illness is widespread. A lot of them would not like to marry a person who is depressed (71.1%), followed by not wanting to work closely with them (45.1%). A similar study found that 49.9% of undergraduate pharmacy students from Nigeria hold the belief that “I would be against any daughter of mine marrying a man who had been to the hospital to see a psychiatrist about mental problems” (Anosike et al., 2020). This study indicated that people are not willing to have in-depth and close contact with patients with depression. The view of keeping social distance may be due to the belief of danger in people with depression, as 60.7% students hold this view in the current study. A line of research studies which also suggest that people with depression (or other mental illness) are dangerous seems to generate increased social distancing (Angermeyer and Matschinger, 2003; Anderson et al., 2015).

### Gender Differences in Stigma Attitude

We found that female students showed a significantly higher willingness to disclose themselves than males if they had this problem described in the vignette, which is in line with previous research (Leong and Zachar, 1999). This gender difference in disclosure willingness may indicate the different genders show a difference in coping styles and attitudes toward seeking professional help (Leong and Zachar, 1999; Brown et al., 2018). This suggested that females are more likely to disclose their own situation and choose to seek help. Another interesting finding is that more female students were willing to work closely with people with depression than male students. A previous study suggested that females are generally more likely to experience stigma about depression or other mental illnesses than males (Farina, 1981; Anderson et al., 2015). A nationwide online survey on a group of UK medical students reported that women showed less stigmatization than men in nearly all conditions, including pneumonia, depression, psychotic symptoms, intravenous drug use, and long-standing unexplained abdominal complaints (Korszun et al., 2012).

### Does Psychiatric Education Play a Role in Reducing Stigma?

In this study, a high proportion of both medical and non-medical students were reported negative attitudes and beliefs toward people with depression. Considering medical students are required to complete psychiatry course, including common mental disorders, such as depression, it is speculated that involving more anti-stigma interventions and mental health literacy into current psychiatric education may help to reduce stigma. The negative attitudes toward depression among medical students might be associated with their “stereotype” that depressive patients are unpredictable, ineloquent, and find it hard to control themselves (Suwalska et al., 2016). A survey on 1,010 Australian-trained medical students reported that higher perceived stigma and higher stress, a past history of anxiety, and Year 3 of medical school were associated with higher personal stigma scores (Cheng et al., 2013). A sample of the Lebanese population showed that higher knowledge of mental illness, high level of education, and being familiar with close people with mental illnesses were associated with less stigmatizing attitudes (Abi Doumit et al., 2019). On the other hand, increased education and experience with mental illnesses are associated with reduced stigma (Sandhu et al., 2019). The high proportion of negative attitudes toward depression may reflect the fact that the present psychiatric education mainly focuses on professional knowledge acquisition, but neglects knowledge about how to reduce mental health stigma and discrimination. The fact also reflects the lack of humanistic and emotional concerns that contributed to reduce stigma and negative attitudes toward depression and other mental disorders. It is imperative to take all available measures to provide more information about depression, which may help reduce the shame about the symptoms.

## Limitation

There are several limitations in the present study. First, the use of convenience sampling in this study may have resulted in inherent deficiencies in sampling, such as poor representativeness and insufficient randomization. Thus, the sample may be biased in some degree and may not be fully representative of the population. However, this study randomly selected different classes by the school, grade, and major. Second, several important sociodemographic factors were not investigated, such as the history of contact with mentally ill patients, receiving psychological counseling, a history of depression or current depression, having important or close people suffering from mental illness, and parents’ education level. Finally, some critical factors (such as racial background, previous contact with someone with depression, and knowledge about mental illness) related to the stigma of depression were not included in the current study.

## Conclusion

The present study found that a high proportion of Chinese college students showed stigma and social distancing from people with depression; male students and medical major students showed a higher stigma in some subscale items toward people with depression. The present results indicate that stigmatization might be a critical interventional target for decreasing depression in university students, and suggest that more anti-stigma interventions should be carried out among college students to reduce their stigma attitudes toward people with depression.

## Data Availability Statement

The raw data supporting the conclusions of this article will be made available by the authors, without undue reservation.

## Ethics Statement

The studies involving human participants were reviewed and approved by the Ethics Committee of the Second Xiangya Hospital of Central South University. Written informed consent for participation was not required for this study in accordance with the national legislation and the institutional requirements.

## Author Contributions

TL: conceived the study. YL and HH: performed the literature review, statistical analyses, and took the lead in writing the manuscript. HH collected the data. YL, QW, YH, and SC revised the manuscript. TL and YL: interpreted the data and commented on the manuscript. All authors contributed to the article and approved the submitted version.

### Conflict of Interest

The authors declare that the research was conducted in the absence of any commercial or financial relationships that could be construed as a potential conflict of interest.

## Footnotes

^1^https://www.ncss.com/
