# Supplementary material for: Stigmatizing Attitudes Toward Depression Among Male and Female, Medical and Non-medical Major College Students
Source: Front Psychol. 2021 Jun 25;12:648059. doi: 10.3389/fpsyg.2021.648059 (PMC8267999; doi:10.3389/fpsyg.2021.648059)
Supplement: Supplementary file 1 [file Table_1.DOCX]

S Tables 1. Percentage of participants who “agree” or “strongly agree” with statements about their own attitudes towards the person in the vignette

| Statement about personal belief (DSS) | Total (N=1056) | |  | Education level | | | | |  | School level | | | | | | |
| --- | --- | --- | --- | --- | --- | --- | --- | --- | --- | --- | --- | --- | --- | --- | --- | --- |
|  | n | % |  | Undergraduates  (n=888) | | Postgraduates  (n=168) | | P |  | National key  (n=463) | | Ordinary  (n=402) | | Three-year college  (n=191) | | P |
|  |  |  |  | n | % (95% CI) | n | % (95% CI) |  |  | n | % (95% CI) | n | % (95% CI) | n | % (95% CI) |  |
| 1. The person could snap out of the problem | 620 | 58.7 |  | 536 | 60.4 (57.2-63.6) | 84 | 50.0 (42.4-57.6) | .012^*^ |  | 247 | 53.3^OJ^ (48.8-57.8) | 246 | 61.2^N^ (56.4-66.0) | 127 | 66.5^N^ (59.8-73.2) | .004^*^ |
| 2. Problem is a sign of personal weakness | 438 | 41.5 |  | 376 | 42.3 (39.1-45.5) | 62 | 36.9 (29.6-44.2) | .190 |  | 188 | 40.6 (36.1-45.1) | 181 | 45.0 (40.1-49.9) | 69 | 36.1 (29.3-42.9) | .106 |
| 3. Problem is not a real medical illness | 316 | 29.9 |  | 263 | 29.6 (26.6-32.6) | 53 | 31.5 (24.5-38.5) | .616 |  | 137 | 29.6 (25.4-33.8) | 116 | 28.9 (24.5-33.3) | 63 | 33.0 (26.3-39.7) | .578 |
| 4. People with this problem are dangerous | 641 | 60.7 |  | 546 | 61.5 (58.3-64.7) | 95 | 56.5 (49.0-64.0) | .229 |  | 274 | 59.2 (54.7-63.7) | 254 | 63.2 (58.5-67.9) | 113 | 59.2 (55.2-66.3) | .432 |
| 5. Avoid people with this problem | 48 | 4.5 |  | 44 | 5.0 (3.6-6.4) | 4 | 2.4 (0.8-4.7) | .142 |  | 32 | 6.9^O^ (4.6-9.2) | 8 | 2.0^N^ (0.6-3.4) | 8 | 4.2 (1.4-7.0) | .002^*^ |
| 6. People with this problem are unpredictable | 308 | 29.2 |  | 249 | 28.0 (25.0-31.0) | 59 | 35.1 (27.9-42.3) | .064 |  | 141 | 30.5 (26.3-34.7) | 116 | 28.9 (24.5-33.3) | 51 | 26.7 (20.4-33.0) | .621 |
| 7. If I had this problem, I wouldn't tell anyone | 96 | 9.1 |  | 83 | 9.3 (7.4-11.2) | 13 | 7.7 (3.67-11.7) | .506 |  | 54 | 11.7^J^ (8.8-14.6) | 32 | 8.0 (5.3-10.7) | 10 | 5.2^N^ (2.1-8.3) | .021^*^ |
| 8. I would not employ someone with this problem | 293 | 27.7 |  | 225 | 25.3 (22.4-28.2) | 68 | 40.5 (33.1-47.9) | .000^*^ |  | 155 | 33.5^J^ (29.2-37.8) | 98 | 24.4 (20.2-28.6) | 40 | 20.9^N^ (15.1-26.7) | .001^*^ |
| 9. I would not vote for a politician with this problem | 448 | 42.4 |  | 363 | 40.9 (37.7-44.1) | 85 | 50.6 (43.0-58.2) | .019^*^ |  | 240 | 51.8^OJ^ (47.2-56.4) | 159 | 39.6^NJ^ (34.8-44.4) | 49 | 25.7^NO^ (19.5-31.9) | .000^*^ |
| Personal stigma score | 26.91±3.56 | |  | 27.05±3.55 | | 26.14±3.70 | | .003^*^ |  | 26.83±4.11 | | 27.05±3.08 | | 26.80±3.21 | | .546 |

Note: N, O, J in column school level represent national key university, ordinary university and junior college respectively. The superscript M represents there is statistic difference compared with medical major. DSS: Personal stigma score of Depression Stigma Scale. Data are n, % or mean ± SD. CI: Confidence Interval *P<0.05.

S Tables 2. Percentage of participants who “agree” or “strongly agree” with statements about other people's attitudes towards the person in the vignette

| Statement about personal belief (DSS) | Total (N=1056) | |  | Education level | | | | |  | School level | | | | | | |
| --- | --- | --- | --- | --- | --- | --- | --- | --- | --- | --- | --- | --- | --- | --- | --- | --- |
|  | n | % |  | Undergraduates  (n=888) | | Postgraduates  (n=168) | | P |  | National key  (n=463) | | Ordinary  (n=402) | | Three-year college  (n=191) | | P |
|  |  |  |  | n | % (95% CI) | n | % (95% CI) |  |  | n | % (95% CI) | n | % (95% CI) | n | % (95% CI) |  |
| 1. The person could snap out of the problem | 626 | 59.3 |  | 571 | 64.3 (61.1-67.5) | 55 | 32.7 (25.6-39.8) | .000^*^ |  | 232 | 50.1^OJ^ (455.5-54.7) | 273 | 67.9^N^ (63.6-72.5) | 121 | 63.4^N^ (56.6-70.2) | <.0001^*^ |
| 2. Problem is a sign of personal weakness | 531 | 50.3 |  | 465 | 52.4 (49.1-55.7) | 66 | 39.3 (31.9-46.7) | .002^*^ |  | 241 | 52.1^J^ (47.5-56.7) | 218 | 54.2 ^J^ (49.3-59.1) | 72 | 37.7^NO^ (30.8-44.6) | .001^*^ |
| 3. Problem is not a real medical illness | 379 | 35.9 |  | 336 | 37.8 (34.6-41.-) | 43 | 25.6 (19.0-32.3) | .002^*^ |  | 158 | 34.1 (29.8-38.4) | 150 | 37.3 (32.6-42.0) | 71 | 37.1 (30.2-44.0) | .572 |
| 4. People with this problem are dangerous | 598 | 56.6 |  | 503 | 56.6 (53.3-59.9) | 95 | 56.5 (49.0-64.0) | .982 |  | 269 | 58.1 (53.6-62.6) | 225 | 56.0 (51.1-60.9) | 104 | 54.5 (47.4-61.6) | .655 |
| 5. Avoid people with this problem | 340 | 32.2 |  | 286 | 32.2 (29.1-35.3) | 54 | 32.1 (25.0-39.2) | .987 |  | 179 | 38.7^OJ^ (34.3-43.1) | 118 | 29.4^N^ (24.9-33.9) | 43 | 22.5^N^ (16.6-28.4) | <.0001^*^ |
| 6. People with this problem are unpredictable | 472 | 44.7 |  | 389 | 43.8 (40.5-47.1) | 83 | 49.4 (41.8-57.0) | .181 |  | 232 | 50.1^OJ^ (455.5-54.7) | 172 | 42.8^N^ (38.0-47.6) | 68 | 35.6^N^ (28.8-42.4) | .002^*^ |
| 7. If I had this problem, I wouldn't tell anyone | 343 | 32.5 |  | 272 | 30.6 (27.6-33.6) | 71 | 42.3 (34.8-49.8) | .003^*^ |  | 178 | 38.4^J^ (34.0-42.8) | 131 | 32.6^J^ (28.0-37.2) | 34 | 17.8^NO^ (12.4-23.2) | <.0001^*^ |
| 8. I would not employ someone with this problem | 525 | 49.7 |  | 420 | 47.3 (44.0-50.6) | 105 | 62.5 (55.2-69.8) | .000^*^ |  | 275 | 59.4^OJ^ (54.9-63.9) | 188 | 46.8^NJ^ (41.9-51.7) | 62 | 32.5^ON^ (25.9-39.1) | <.0001^*^ |
| 9. I would not vote for a politician with this problem | 590 | 55.9 |  | 477 | 53.7^M^ (50.4-57.0) | 113 | 67.3 (60.2-74.4) | .001^*^ |  | 299 | 64.6^OJ^ (60.2-69.0) | 222 | 55.2^NJ^ (50.3-60.0) | 69 | 36.1^ON^ (29.3-42.9) | <.0001^*^ |
| Perceived stigma score | 29.62±4.83 | |  | 29.78±4.824 | | 28.74±4.799 | | .011^*^ |  | 29.89±4.72 | | 29.97±4.96 | | 28.20±4.61 | | .000^*^ |

Note：N, O, J in column school level represent national key university, ordinary university and junior college respectively. DSS: Perceived stigma score of Depression Stigma Scale. Data are n, % or mean ± SD. CI: Confidence Interval *P<0.05. Data are n, % or mean ± SD. CI: Confidence Interval *P<0.05

S Table 3. Percentage of participants who “agree” or “strongly agree” with statements about other people's attitudes towards the person in the vignette

| Statement about personal belief (DSS) | Total (N=1056) | |  | Gender | | | | |  | Major | | | | |
| --- | --- | --- | --- | --- | --- | --- | --- | --- | --- | --- | --- | --- | --- | --- |
|  | n | % |  | Male (n=355) | | Female (n=701) | | P |  | Non-Medical (n=752) | | Medical (n=304) | | P |
|  |  |  |  | n | % (95% CI) | n | % (95% CI) |  |  | n | % (95% CI) | n | % (95% CI) |  |
| 1. The person could snap out of the problem | 626 | 59.3 |  | 203 | 57.2 (52.1-62.3) | 423 | 60.3 (56.7-63.9) | .324 |  | 453 | 60.2 (56.7-63.7) | 173 | 56.9 (51.3-62.5) | .318 |
| 2. Problem is a sign of personal weakness | 531 | 50.3 |  | 181 | 51.0 (45.8-56.2) | 350 | 49.9 (46.2-53.6) | .745 |  | 348 | 46.3 (42.7-49.9) | 183 | 60.2 (54.7-65.7) | <.0001^*^ |
| 3. Problem is not a real medical illness | 379 | 35.9 |  | 147 | 41.4 (36.3-46.5) | 232 | 33.1 (29.6-36.6) | .008^*^ |  | 283 | 37.6 (34.1-41.1) | 96 | 31.6 (26.4-36.8) | .063 |
| 4. People with this problem are dangerous | 598 | 56.6 |  | 177 | 49.9 (44.7-55.1) | 421 | 60.1 (56.5-63.7) | .002^*^ |  | 428 | 56.9 (53.4-60.4) | 170 | 55.9 (50.3-61.5) | .768 |
| 5. Avoid people with this problem | 340 | 32.2 |  | 109 | 30.7 (25.9-25.5) | 231 | 33.0 (29.5-36.5) | .460 |  | 226 | 30.1 (26.8-33.4) | 114 | 37.5 (32.1-42.9) | .019^*^ |
| 6. People with this problem are unpredictable | 472 | 44.7 |  | 146 | 41.1 (36.0-46.2) | 326 | 46.5 (42.8-50.2) | .097 |  | 320 | 42.6 (39.1-46.1) | 152 | 50.0 (44.4-55.6) | .028^*^ |
| 7. If I had this problem, I wouldn't tell anyone | 343 | 32.5 |  | 106 | 29.9 (25.1-34.7) | 237 | 33.8 (30.3-37.3) | .195 |  | 234 | 31.1 (27.8-34.4) | 109 | 35.9 (30.5-41.3) | .137 |
| 8. I would not employ someone with this problem | 525 | 49.7 |  | 171 | 48.2 (43.0-53.4) | 354 | 50.5 (46.8-54.2) | .474 |  | 346 | 46.0 (42.4-49.6) | 179 | 58.9 (53.4-64.4) | <.0001^*^ |
| 9. I would not vote for a politician with this problem | 590 | 55.9 |  | 186 | 52.4 (47.2-57.6) | 404 | 57.6 (53.9-61.3) | .105 |  | 389 | 51.7 (48.1-55.3) | 201 | 66.1 (60.8-71.4) | <.0001 ^*^ |
| Perceived stigma score | 29.62±4.83 | |  | 29.51±5.095 | | 29.67±4.698 | | .610 |  | 29.40±4.79 | | 30.14±4.90 | | .024^*^ |

DSS: Perceived stigma score of Depression Stigma Scale. Data are n, % or mean ± SD. CI: Confidence Interval *P<0.05

S Tables 4. Percentage of participants who “probably unwilling” or “definitely unwilling” to have contact with the person described in the vignette

| Statement about  personal belief (SDS) | Total (N=1056) | |  | Education level | | | | |  | School level | | | | | | |
| --- | --- | --- | --- | --- | --- | --- | --- | --- | --- | --- | --- | --- | --- | --- | --- | --- |
|  | n | % |  | Undergraduates  (n=888) | | Postgraduates  (n=168) | | P |  | National key  (n=463) | | Ordinary  (n=402) | | Three-year college (n=191) | | P |
|  |  |  |  | n | % (95% CI) | n | % (95% CI) |  |  | n | % (95% CI) | n | % (95% CI) | n | % (95% CI) |  |
| 1. Live next door | 289 | 27.4 |  | 245 | 27.6 (24.7-30.5) | 44 | 26.2 (19.6-32.8) | .709 |  | 150 | 32.4^OJ^ (28.1-36.7) | 99 | 24.6^N^ (20.4-28.8) | 40 | 20.9^N^ (15.1-26.7) | .003^*^ |
| 2. Spend the evening socializing | 208 | 19.7 |  | 172 | 19.4 (16.8-22.0) | 36 | 21.4 (15.2-27.6) | .538 |  | 111 | 24.0^OJ^ (20.1-27.9) | 68 | 16.9^N^ (13.2-20.6) | 29 | 15.2^N^ (10.1-20.3) | .008^*^ |
| 3. Make friends | 253 | 24.0 |  | 219 | 24.7 (21.9-27.5) | 34 | 20.2 (14.1-26.3) | .218 |  | 121 | 26.1 (22.1-30.1) | 94 | 23.4 (19.3-27.5) | 38 | 19.9 (14.2-25.6) | .222 |
| 4. Work closely | 476 | 45.1 |  | 405 | 45.6 (42.3-48.9) | 71 | 42.3 (34.8-49.8) | .424 |  | 225 | 48.6 (44.0-53.2) | 170 | 42.3 (37.5-47.1) | 81 | 42.4 (35.4-49.4) | .127 |
| 5. Marry into family | 751 | 71.1 |  | 634 | 71.4 (68.4-74.4) | 117 | 69.6 (62.6-76.6) | .646 |  | 333 | 71.9 (67.8-76.0) | 291 | 72.4 (68.0-76.8) | 127 | 66.5 (59.8-73.1) | .294 |
| Social distance score | 11.50±2.76 | |  | 11.57±2.68 | | 11.11±3.13 | | .074 |  | 11.59±2.86 | | 11.49±2.76 | | 11.28±2.52 | | .417 |

Note: N, O, J in column school level represent national key university, ordinary university and junior college respective. SDS: Social Distance Scale. Data are n, % or mean ± SD. CI: Confidence Interval*P<0.05
